# Semi-Automatic Artificial Lips Device for Brass Instruments with Real-Time Pitch Feedback Control

**DOI:** 10.3390/s26030984

**Published:** 2026-02-03

**Authors:** Hiroaki Sonoda, Hikari Kuriyama, Kouki Tomiyoshi, Gou Koutaki

**Affiliations:** 1 Graduate School of Science and Technology, Kumamoto University, Kumamoto 860-8555, Japan; sonoda@navi.cs.kumamoto-u.ac.jp (H.S.); kuriyama@navi.cs.kumamoto-u.ac.jp (H.K.); tomiyoshi@navi.cs.kumamoto-u.ac.jp (K.T.); 2 Department of Computer Science and Electrical Engineering, Kumamoto University, Kumamoto 860-8555, Japan

**Keywords:** acoustic sensing, brass instrument, artificial lips, feedback control

## Abstract

We propose a semi-automatic artificial lips control device that allows a human performer to produce sound on a brass instrument without the need to vibrate their own lips. The device integrates position control that presses artificial lips toward the mouthpiece and aperture control via wire traction, together with a pre-calibrated motor table and acoustic feedback for pitch stabilization. In evaluations using a euphonium, we verified timbre, pitch range, and pitch stabilization, including harmonic modes. The results showed that the harmonic structure of tones produced by a human using the device can be comparable to those produced by a human player in the conventional manner. Pitch-range and pitch-stabilization tests confirmed that the system can generate practical musical intervals and achieve reliable harmonic mode changes. Furthermore, real-time acoustic feedback improved pitch stability during performance. These findings demonstrate that, rather than fully automating human performance, the proposed system provides a compact and reproducible framework for controllable brass sound generation and pitch stabilization using only three actuators.

## 1. Introduction

Musical performance robots and artificial blowing devices for wind instruments have been developed for various purposes. The development of these robots plays a role across various fields, including applications in music education and performance support [1,2], aiming to replace human performers or create new musical expressions [3], and music therapy [4]. Automated musical instruments have been realized for a wide range of instrument types, such as pianos, percussion, and string instruments [5,6,7,8]. In particular, automatic blowing devices for brass instruments have been widely used for modeling lip vibration, clarifying sound production mechanisms, and experimentally validating blowing sounds using artificial lips [9,10,11,12,13,14,15,16,17]. However, performing the entire complex sound production process of brass instruments in a fully automated manner requires the control of artificial lips, artificial teeth, an artificial oral cavity, and an air-supply system. Such systems make the device large-scale, which poses problems with maintenance and reproducibility, making it unsuitable for casual acoustic experiments or performances.

To solve this problem, this study proposes a semi-automated blowing device with a structure simplified as much as possible. The proposed device features a compact mounting mechanism and an artificial lips control system, enabling convenient use for analyzing the acoustic characteristics of brass instruments and collecting realistic sound production data.

To clarify the scope and contribution of this study, the key points are summarized as follows:

- Purpose: To enable controllable and reproducible sound production on brass instruments through partial automation, without replacing human performers.
- Intended application: Compact acoustic experiments, performance support, and the construction of semi-automatic brass performance systems that enable controlled playing of real acoustic instruments without requiring full automation.
- System overview: The proposed system produces sound when a performer blows air into a brass instrument equipped with an artificial lips control device, causing the artificial lips to vibrate and generate a buzzing excitation. The vibration behavior is modified to vary and stabilize pitch through real-time acoustic feedback.

This section provides an overview of the sound production mechanism of brass instruments and previous developments of automatic blowing devices, followed by the basic concept and position of this study.

### 1.1. Brief Description of Brass Instruments

**Figure 1:**
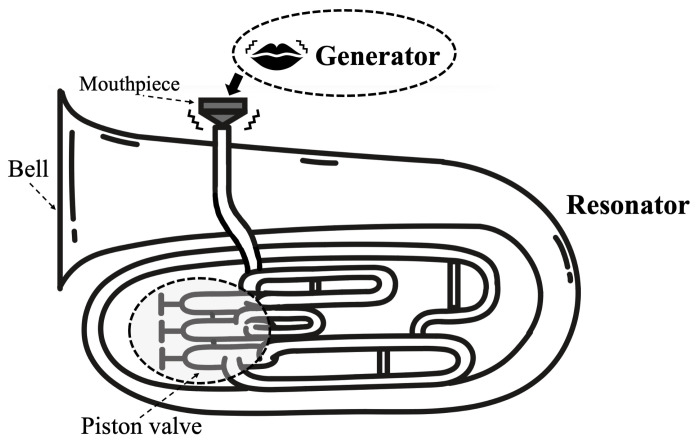
Euphonium illustration. The vibration of the lip reed is transmitted to the resonator via the mouthpiece. The resonant frequency can be changed by pressing the piston valve.

This section outlines the principles of sound production and pitch variation in brass instruments. Sound in wind instruments arises from self-sustained oscillation, produced through the interaction between a “Generator” that initiates vibration and a “Resonator” that provides acoustic feedback [18]. In brass instruments, the player’s lips function as the generator, and their vibration is transmitted through the mouthpiece to the air column. The target instrument in this study, the euphonium (Figure 1), is a mid-low brass instrument with a wide conical bell. Its resonance frequencies can be altered by extending the tube length using three or four piston valves.

#### 1.1.1. Generator

At the generator, vibration is produced by placing the lips on the mouthpiece and blowing air into the instrument. The acoustic pressure reflected from the bell forms a closed feedback loop, allowing the lips to continue vibrating. Previous studies have shown that the lip vibration frequency generally matches the resonance frequency of the air column during steady oscillation [13]. Ehara et al. [11] reported, based on artificial lips experiments and numerical analysis, that the excited resonance mode shifts to a higher mode as the natural frequency of the lips increases, while thicker lips suppress higher-mode excitation.

Performers adjust the natural frequency of the lips by controlling facial muscles and the relative lip position on the mouthpiece. This control, known as the embouchure, effectively tunes the natural frequency of the lip oscillator.

Numerous models have been proposed to describe how lip vibration frequency changes. Adachi et al. [19] modeled the lips as a two-dimensional mass–spring system. Strauss et al. [20] modeled the lips as a semicircular thin vibrating plate, assuming that the vibrational characteristics depend on the contact condition between the lips and the mouthpiece. This model, referred to as the Farkas–Arban–Leno model, treats the deformation modes of the lips as an eigenvalue problem, in which the dimensionless eigenvalue λ is determined by the boundary condition at the rim of the mouthpiece.

**Figure 2:**
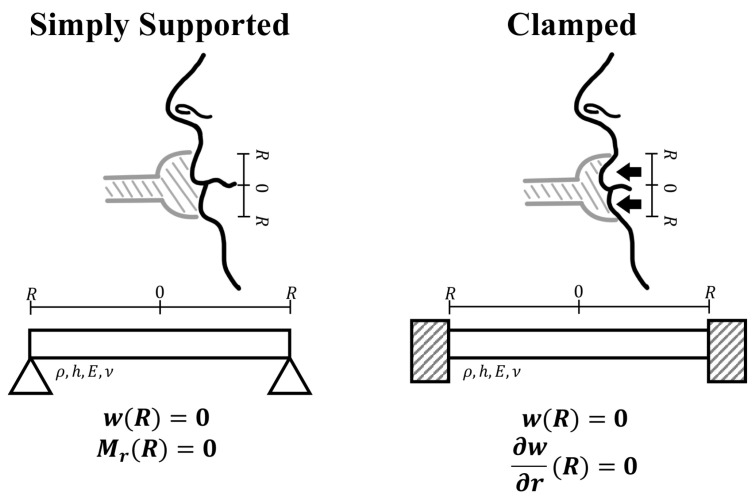
Relationship between boundary conditions of lip vibration model and brass instrument performance. The lips are clamped by forcing them into the mouthpiece.

As illustrated in Figure 2, a simply supported boundary represents a condition in which the lips are lightly in contact with the mouthpiece rim, allowing rotational freedom. In this case, the radial displacement at the lip edge is constrained to zero (w(R)=0), while the radial component of the bending moment vanishes ($M_{r}\left(R\right)=0$). In contrast, a clamped boundary corresponds to a condition in which the lips are pressed firmly against the mouthpiece, constraining both displacement and slope. Accordingly, both the displacement and its radial derivative are fixed at the boundary, expressed as w(R)=0 and ∂w/∂r(R)=0.

Assuming a Poisson’s ratio of ν=0.49 to account for the near-incompressibility of lip tissue [21], Strauss et al. numerically computed the eigenvalues corresponding to each boundary condition. Their results showed that, for the lowest vibration mode, transitioning from a simply supported to a clamped boundary approximately doubles the squared eigenvalue $\lambda^{2}$.

For the lowest mode, the natural angular frequency $\omega_{0}$ and the natural frequency $f_{0}$ of the lips are expressed as follows:

(1) $$f_{0}=\frac{\omega_{0}}{2\pi }=\frac{\lambda^{2}h}{2\pi R^{2}}\sqrt{\frac{E}{12\rho (1-\nu^{2})}}\propto \lambda^{2}$$

Here, *h* denotes the lip thickness, *R* the inner radius of the mouthpiece, *E* Young’s modulus, ρ the material density, and ν Poisson’s ratio.

Thus, if all other parameters are constant, doubling $\lambda^{2}$ results in doubling the natural frequency, corresponding to a one-octave rise in pitch. Although human performers can produce a wider frequency range through muscular control, the device proposed in this study focuses on pitch variation induced by changing the boundary condition, achieved by pressing the artificial lips into the mouthpiece (Section 2.2.3).

#### 1.1.2. Resonator

The resonator amplifies the vibration introduced at the mouthpiece and adjusts the effective tube length so that its resonance mode matches the lip vibration mode. Changes in lip vibration allow the production of harmonic tones—integer multiples of the fundamental frequency (the pedal tone)—but intermediate pitches require modifying the tube length. Instruments such as the euphonium and trumpet use piston valves for this purpose. Pressing a piston reroutes the airflow through additional tubing, increasing the tube length and lowering the resonance frequency. Except for the pedal tone, the interval between the second (C) and third (G) modes is the widest and includes six scale tones, which can be played by combining the three-valve system [22]. In this study, only the generator is automated, and valve operation is performed by the player.

### 1.2. Basic Ideas

To automate brass sound production, previous artificial blowing systems [3] have used artificial lips whose position and tension were actively controlled. However, these systems typically require multiple components, including air-supply mechanisms and artificial lips assemblies, which makes the overall setup large, complex, and difficult to operate.

**Figure 3:**
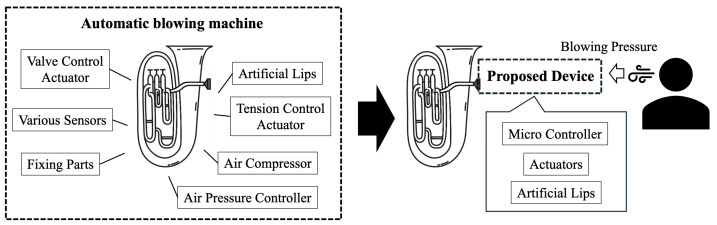
Basic idea. Conventional automatic blowing devices require various parts and complex controls. The device proposed in this study is small and has a simple design, allowing sound production by a performer blowing air into the instrument, making it easy for anyone to make sounds.

To overcome these limitations, this study proposes a semi-automatic device that enables artificial lips control while maintaining a simple and portable design (Figure 3). The device aims to enable users to produce brass sounds by blowing air into the instrument, even without prior brass-playing experience, through partial automation of the sound generation mechanism. By minimizing the hardware complexity, the device is easy to attach, remove, and carry. In addition, acoustic information during playing is sensed in real time and used for feedback correction of the artificial lips, enabling more stable sound production.

Air-pressure blowing systems are typically bulky and offer limited maintainability and repeatability. Thus, we adopt a “semi-automatic” design in which the performer supplies the airflow, allowing their breath to excite the artificial lips and produce sound.

### 1.3. Technical Overview

We developed the following technical components in this system.

(a) Simple and easy-to-use blowing device
  The device is designed to be simple and easy to use, requiring only attachment to the mouthpiece. To avoid the bulkiness of previous systems, no pneumatic pump is used; the performer provides the airflow.
(b) Artificial lips that anyone can create
  As in prior studies, sound is generated by exciting an artificial lips structure. For reproducibility and ease of fabrication, the artificial lips are molded from readily available urethane gel.
(c) Position control of artificial lips using a servo motor
  We developed a system in which a motor controls the position of artificial teeth, which indirectly pushes the artificial lips. This approach leverages the fact that pressing the lips toward the mouthpiece enables pitch variation. Using a servo motor offers advantages such as low operating voltage, low heat generation, and accurate positioning based on commanded rotation angles.
(d) Aperture control by fundamental frequency (f0) feedback
  In the proposed device, motor control is based on pre-calibrated data, complemented by real-time acoustic sensing during performance. Feedback is applied to selected motor actions using the detected fundamental frequency (f0). Real-time pitch sensing is particularly effective for nonlinear robotic performers.

### 1.4. Related Works

This section discusses previous artificial playing systems for brass instruments.

#### 1.4.1. Blowing Device Using Artificial Lips

Various materials and structural designs have been explored for artificial lips in such systems. Major examples are as follows.

Water-filled latex tubes: Gilbert et al. [9] and Teissier et al. [10] developed artificial lips using water-filled latex tubes. The embouchure is adjusted by controlling the tube tension and the relative position between the lips and the mouthpiece. These systems successfully generate reed-like lip vibrations similar to those of human players. The same concept was later applied to a trombone-playing device by Lopes et al. [15].

Gel sheets: Kaneko et al. [12] used gel sheets fixed over an aluminum plate to generate artificial lip vibration. The lip–mouthpiece distance is controlled using a lip-slide mechanism, with the aluminum plate ensuring smooth sliding motion.

Silicone: Ehara et al. [11] conducted blowing experiments using silicone sheets with a central air hole for the measurement and modeling of artificial lips. In recent years, Fréour et al. [16,17] developed the pocket artificial buzzing (PAB) system, which produces lip reed vibrations using two semicircular silicone sheets. Unlike other systems, the PAB is not automated. By removing actuators for embouchure adjustment, the device becomes compact and portable. When attached to an instrument, it allows users to obtain its acoustic characteristics with minimal adjustment.

#### 1.4.2. Robot Sensing Using Acoustic Feedback

Among various musical performance robots, feedback control based on performance information is often used when feedforward actuator control is difficult or when stable performance is required.

Mizumoto et al. [6] developed a robot that plays the theremin, an electronic instrument with two antennas controlling pitch and volume. Because the theremin exhibits nonlinear pitch characteristics, the system combines parametric feedforward control with feedback based on the detected pitch. An autocorrelation-based method is used for pitch estimation, and PI control is applied to reduce pitch errors. Hanai et al. [7] developed a robot for the musical saw, a body-resonance instrument whose acoustic characteristics vary with temperature, humidity, and friction. They implemented pitch-based feedback control and determined the striking position using peak detection in the FFT results and proportional control. Tokarczyk et al. [8] used acoustic feedback to control a robotic arm that plays the guitar. By adjusting motor coordinates according to the produced sound level, stable performance is maintained even when the instrument setup or pick position changes.

### 1.5. Position of This Study

**Figure 4:**
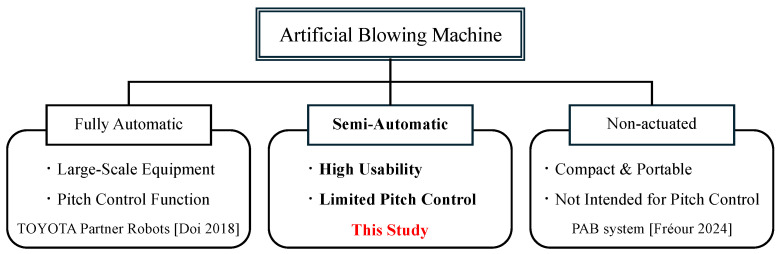
Classification of automatic blowing devices. This figure illustrates the position of the system proposed in this study. Unlike fully automatic robot [3] and non-actuated devices [16], this system achieves limited pitch control—the production of pitches within a restricted range of the instrument—through motor control while maintaining high usability.

The position of this study is illustrated in Figure 4. The goal of this research is not to replace human performers with a fully automated system, but to enable controllable and reproducible brass sound production through partial automation, even for users without prior brass-playing experience. While artificial lips actuation allows for limited pitch control within the range of the instrument, the system is intentionally kept simple to preserve usability. Thus, the proposed device lies between non-automated systems [16] and fully automated blowing systems [3], providing a compact and semi-automatic alternative. Rather than pursuing full automation, this intermediate approach constitutes the novelty of the present study by demonstrating that essential aspects of brass sound generation and pitch control can be achieved with a simplified actuator configuration.

Simply removing the air-supply system from existing fully automatic devices would not meet our objectives. The device must be compact, low-heat, and noninvasive, and it must allow stable sound production using only the performer’s breath pressure. Easy attachment and detachment from the instrument are also required to maintain usability.

Previous studies have shown that the most effective parameter for controlling the embouchure is the relative position between the mouthpiece and the lips [9]. Based on this finding, the proposed system adopts a simplified control strategy in which the artificial lips are pushed toward the mouthpiece.

## 2. Proposed System

This section describes the configuration and control system of the proposed semi-automatic device.

### 2.1. Overview of the Proposed System

**Figure 5:**
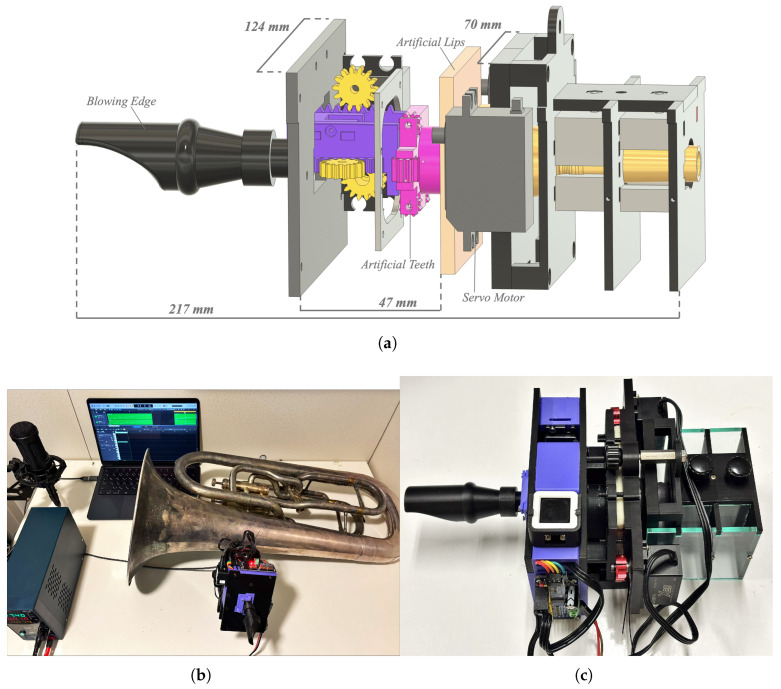
Overall view of the proposed system. (**a**) CAD diagram showing the internal structure. (**b**) Euphonium equipped with the device and the surrounding environment during operation. (**c**) Close-up photograph of the proposed device.

An overview of the proposed device is shown in Figure 5. The device consists of three main components: the attachment unit, the oral unit, and the control unit. The structure is made of acrylic plates and PLA parts printed from 3D CAD models, meeting the design requirements of compactness and low weight (total weight: 605 g). The actuators consist of three servo motors (GXServo X25, Dongguan Gongxun Power Technology Co., Ltd., Dongguan, China), controlled by an ATOM S3 microcontroller (M5Stack Technology Co., Ltd., Shenzhen, China) and a PCA9685 motor driver (NXP Semiconductors, Eindhoven, The Netherlands).

### 2.2. Mechanical Structure

**Figure 6:**
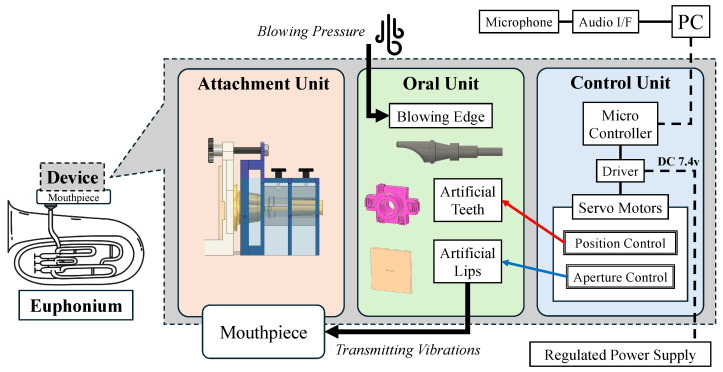
Hardware configuration diagram. The device has a compact design consisting of 3 basic elements: Attachment Unit, Oral Unit, and Control Unit. It is attached to the mouthpiece and the performer can play the instrument by blowing into the device.

The hardware configuration of the proposed device is shown in Figure 6.

#### 2.2.1. Attachment Unit

The attachment unit is designed to be mounted to the mouthpiece. To simplify installation and removal, the device is fixed to the mouthpiece using two M3 bolts rather than being attached directly to the instrument body.

#### 2.2.2. Oral Unit

**Figure 7:**
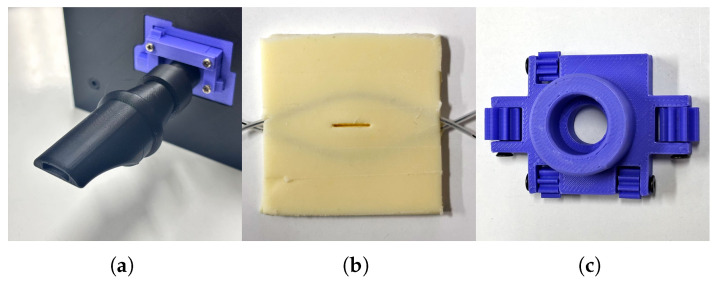
Components of the oral unit. (**a**) The blowing edge where the performer blows. (**b**) Artificial lips. (**c**) Artificial teeth used to control the position of the artificial lips.

The oral unit consists of three subcomponents (Figure 7).

Blowing Edge: The performer blows into the mouthpiece without vibrating their own lips. Because the device does not include any air-supply system, the blowing edge is shaped like that of a recorder to ensure sufficient airflow for sustaining artificial-lip vibration.

Artificial Lips: The artificial lips are made from Asker C0 urethane gel (Exseal Co., Ltd., Mino City, Gifu, Japan). During molding, a rectangular aperture is formed, and a wire covered with silicone tubing is embedded around it. This mimics the function of the human orbicularis oris muscle: pulling the wire from both sides applies load toward the aperture and adjusts the lip opening. The artificial lips are mounted to a 3D-printed holder, allowing easy removal.

Artificial Teeth: The artificial teeth adjust the degree to which the artificial lips are pushed toward the mouthpiece. They have a cylindrical shape and move along a guide within an enclosed chamber. A gear mechanism is used for the guide to absorb dimensional errors inherent in 3D-printed parts.

#### 2.2.3. Control Unit

The artificial lips are controlled using two mechanisms: position control, in which the artificial teeth are pushed toward the mouthpiece, and aperture control, in which the internal wire is tensioned. Power to the servo motor is supplied via a regulated power supply (7.4 V). During use, the motor is rotated to the angle corresponding to the target pitch. To transmit this target pitch data and the frequency-difference data described later, the PC is connected to the microcontroller.

**Figure 8:**
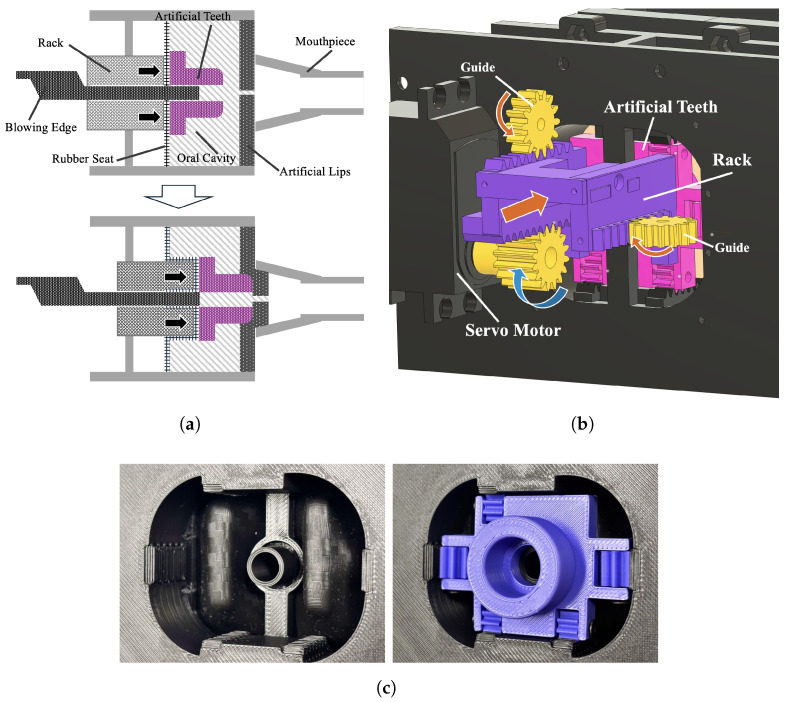
Artificial lips “Position Control” mechanism. To maintain a tight seal, the rack presses the artificial teeth from the outside of the rubber membrane. (**a**) Schematic diagram of position control. (**b**) CAD drawing of the motor and rack during control operation. (**c**) Photograph of the oral cavity and artificial teeth when actually in operation.

Position Control: The position control unit (Figure 8) adjusts the relative position between the artificial lips and the mouthpiece. By pushing the rack toward the mouthpiece, the artificial teeth are indirectly pressed via a rubber membrane, changing the lip-pushing depth. One servo motor is used, and a rack-and-pinion mechanism converts motor rotation into linear motion. As the rack and the artificial teeth move along the guide, the displacement can be controlled by specifying the motor’s rotation angle. The motor angle is controlled by sending PWM (Pulse Width Modulation) signals from the microcontroller. PWM is a technology that controls signals and power by changing the ratio of the on-time and off-time of a pulse signal.

**Figure 9:**
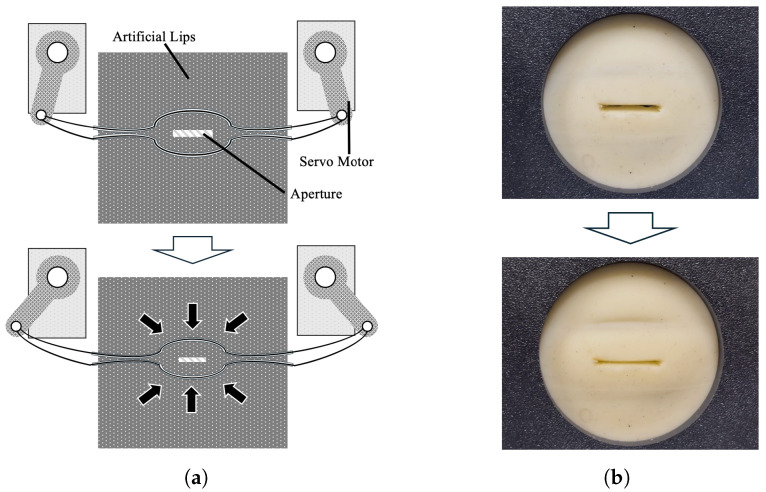
“Aperture Control” mechanism. The side motors attached to the wires rotate to tighten the air holes, applying tension. (**a**) Schematic diagram of applying tension to the aperture. (**b**) Photograph of artificial lips with tension applied.

Aperture Control: The aperture control unit (Figure 9) applies load toward the lip aperture by pulling the internal wire from both sides. Two servo motors are used; the wire is fixed to each motor’s swing arm and tensioned by rotation. As in the position control unit, the aperture is adjusted by specifying the motor rotation angles.

### 2.3. System Configuration and Control Method

**Figure 10:**
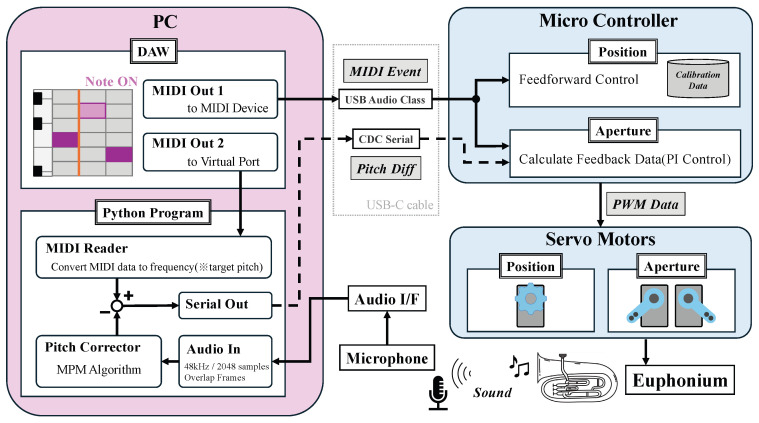
Control system diagram. The motor is operated based on MIDI messages sent from the PC. At the same time, a message is sent to the Python program, which calculates the frequency difference with the estimated playing sound and feeds it back to the microcontroller. The rotation angle of the aperture control motor is corrected based on this difference data.

Figure 10 shows the overall control structure. The microcontroller adjusts the position and aperture of the artificial lips by controlling three servo motors based on MIDI data and pitch feedback signals (frequency-difference data) received from a PC.

On the PC, the target pitch is generated in a DAW (Logic Pro) and sent as MIDI data. Logic Pro transmits the MIDI signal to both the microcontroller (as an external MIDI device) and a virtual MIDI port used by a Python program for capturing MIDI events. The MIDI data are sent to the microcontroller via a USB-C cable and simultaneously sent to a Python program via a virtual MIDI port, which calculates the difference (Pitch Diff) between the f0 frequency and the target pitch. Here, the conversion from the note number in the MIDI data to the reference pitch is implemented by converting the note number to frequency based on 12-tone equal temperament with A4 = 440 Hz as the reference.

The Pitch Diff is transmitted to the microcontroller via USB serial communication, where it is used to update the motor angle commands. For safety, an upper limit is imposed on the PWM values sent to the motors to ensure that they do not reach stall torque or apply excessive force to the artificial lips. The Pitch Diff computation program was developed in Python 3.11.11 using VS Code. A single script integrates audio processing for pitch estimation, MIDI input, and serial communication with the microcontroller for experimental implementation.

The microcontroller drives three servo motors via I^2^C communication with the PCA9685 motor driver: one motor for position control of the artificial teeth and two for aperture control of the artificial lips.

#### 2.3.1. Artificial Lips Position Control

The distance between the artificial lips and the mouthpiece is adjusted by controlling the forward displacement of the artificial teeth. Advancing the teeth pushes the artificial lips against the mouthpiece, shifting the boundary condition from a loose state to a clamped state, resulting in a change in the produced pitch.

This study assumes that the pitch increases monotonically with pushing depth and constructs a lookup table through prior calibration. Calibration is performed while actually producing sound, and the position chosen is not the one closest to the ideal pitch, but the one at which the onset of oscillation is most stable. Using this lookup table, the motor can be moved immediately to the position corresponding to any target pitch.

#### 2.3.2. Aperture Control with a PI Controller

Position control alone cannot ensure correct pitch production owing to disturbances such as material nonlinearity, hysteresis, humidity, and degradation. Additionally, hysteresis during harmonic transitions has also been reported.

To address these issues, this study implements a feedback system that senses acoustic information in real time, computes the difference between the detected fundamental frequency (f0) and the target pitch, and adjusts the motor angle accordingly. This allows the system to adapt to environmental changes and maintain stable sound production. Specifically, the Pitch Diff, defined as the difference between the target pitch and the sensed f0, is computed and transmitted to the microcontroller via USB serial communication. The microcontroller then modifies the aperture-control motor angle based on this value. The system uses classical PI control, as expressed in Equations (2) and (3). Here, $P_{Target}$ denotes the target pitch derived from the MIDI note number, and $P_{Estimate}$ represents the estimated pitch obtained from the audio signal. The error signal e(τ) corresponds to the Pitch Diff, u(t) is the control output corresponding to the PWM pulse-width correction, $K_{p}$ and $K_{i}$ are the proportional and integral gains, respectively, and *t* and τ denote time.

(2) $$e\left(\tau \right)=P_{Target}-P_{Estimate},$$

(3) $$u\left(t\right)=K_{p}e\left(t\right)+K_{i}\int_{0}^{t}e\left(\tau \right)d\tau .$$

Large pitch fluctuations may occur during brass instrument playing due to octave jumps and other mode transitions, which can cause abrupt changes in the pitch-estimation result. To ensure safe operation, a PID controller including a derivative term was not used, because rapid fluctuations in the estimated pitch can induce excessively sensitive motor responses, thereby increasing mechanical stress and compromising operational safety.

### 2.4. Data Processing and Analysis

This section describes the detection of the fundamental frequency used for control, the transmission of MIDI messages for the target pitch, and the communication process between the PC and the device.

#### 2.4.1. Fundamental Frequency Estimation

To estimate the fundamental frequency (f0) corresponding to the played pitch, this study employs the McLeod Pitch Method (MPM), a periodicity-based autocorrelation approach [23]. In general, f0-estimation methods are classified into signal-processing-based and machine-learning-based approaches. For real-time feedback control in robotic systems, signal-processing methods are preferred due to their low computational cost and fast response. Several time-domain periodicity detectors exist, including YIN [24] and SWIPE, which has also been used in a theremin-playing robot [25]. MPM was chosen because it offers low computational cost, high temporal resolution, and robustness against octave errors. The procedure for estimating f0 using MPM is as follows.

The autocorrelation function (ACF) of the input signal is defined in Equation (4), where $x_{t}$ is the sampled input signal, *W* is the frame size, *t* is the start index, and τ is the candidate time delay for the f0 period.

(4) $$ACF:r_{t}\left(\tau \right)=\sum_{j=t}^{t+W-1-\tau }x_{j}x_{j+\tau }$$

The squared difference function (SDF) is defined in Equation (5), and the normalized squared difference function (NSDF) is defined in Equation (6). The f0 is obtained by locating the local maxima of the NSDF.

(5) $$SDF:d_{t}\left(\tau \right)=\sum_{j=t}^{t+W-1-\tau }{\left(x_{j}-x_{j+\tau }\right)}^{2},$$

(6) $$NSDF:n_{t}^{\prime }\left(\tau \right)=\frac{2\sum_{j=t}^{t+W-1-\tau }x_{j}x_{j+\tau }}{\sum_{j=t}^{t+W-1-\tau }\left(x_{j}^{2}+x_{j+\tau }^{2}\right)}.$$

In this system, audio data are captured in real time using PyAudio v0.2.14 at a 48 kHz sampling rate and a frame length of W=2048 sample. Because the NSDF requires scanning τ from 0 to W−1, the sampling rate and frame size affect the temporal resolution. With these parameters, the resulting temporal resolution is approximately 42.7 ms. In this study, the pitch correction program does not aim to respond to rapid, millisecond-scale pitch fluctuations; therefore, this temporal resolution was considered sufficient.

#### 2.4.2. Handling MIDI Messages

MIDI (Musical Instrument Digital Interface) is a standard for transmitting performance information—pitch, velocity, timing, and other parameters—between electronic instruments and software [26,27]. DAWs can send MIDI messages to connected external devices. In the proposed system, the DAW sends MIDI messages to the microcontroller, which extracts Note-On events and uses the corresponding pitch to retrieve the calibrated motor angle from the lookup table. For example, when a Note-On message for D3 is sent, the microcontroller selects the calibrated angle for D3 and moves the motor to produce the corresponding pitch.

The microcontroller supports USB-OTG, allowing it to be recognized as an external MIDI device via the USB Audio Class. While pitch-difference data for aperture control are transmitted via USB Serial, both communication protocols coexist on a single USB cable, with the microcontroller functioning as a composite device.

## 3. Evaluation Experiment

### 3.1. Experimental Methods

We conducted the following three experiments and evaluations to evaluate the effectiveness of the device we developed.

**Exp. 1—Timbre Analysis:** Normal (without the device) vs. device-assisted playing
**Exp. 2—Pitch Range Test:** Pitch variation achievable with “Position Control” only
**Exp. 3—Pitch Stabilization Test:** Pitch transition behavior using “Aperture Control”

In Experiment 1, the same pitch (A♯3) was produced in two conditions: (i) normal playing (without the device) and (ii) blowing into the instrument with the device attached. All performances were carried out by the author. Because the proposed device assumes that the user provides airflow, we also examined whether articulation can be achieved through breath pressure. Articulation refers to expressive techniques that shape the connection between notes, such as creating crescendos by varying the blowing intensity.

Experiment 2 evaluates how much pitch variation can be achieved using position control alone, without aperture control. The PWM pulse width of the position-control motor was varied in steps of 10, and for each motor angle, the performer pressed the pistons corresponding to all fingering patterns FG1–FG7 (Table 1). A 10-step increase in PWM corresponds to a forward displacement of +0.5 mm for the artificial teeth. For each condition, the performer blew with the minimum pressure necessary to produce sound, and the resulting pitch was recorded. Peak-frequency identification for each produced sound was performed using the TonalEnergy tuner, a widely used commercial tuner application with a pitch display resolution of 0.1 cent. Only one formal measurement was conducted per condition because preliminary tests confirmed that the boundary between oscillating and non-oscillating states was highly stable, and detecting small variations was not the objective of this experiment.

**Figure 11:**
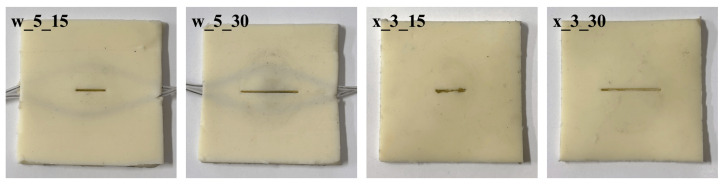
Artificial lips created by changing parameters.

Four types of artificial lips with different thicknesses and mechanical properties (Figure 11, Table 2) were used in Exp. 1 and 2 to investigate how lip characteristics affect sound production. Because the embedded silicone-tube wire cannot be inserted into thinner lip samples, the 3 mm thick lips were not equipped with the internal wire.

In Experiment 3, we investigate whether aperture control can be used to stably play at a pitch close to the target pitch. Motor calibration was first performed within the pitch range playable through position control, following the procedure described in Section 2.3.1. Then, the system was driven by MIDI messages to execute the specified pitch transitions. During playing, aperture feedback control was applied, and the time-series data of the produced pitch were recorded. The performer also applied the corresponding fingerings for each target pitch. The artificial lips used were of type w_5_15. The performance sequence consisted of repeating the ascending pattern F3 → G3 → A3 → A♯3. Because F3 and A♯3 share the same fingering (FG1: open), producing this pattern requires the artificial lips to jump between harmonic modes.

**Figure 12:**
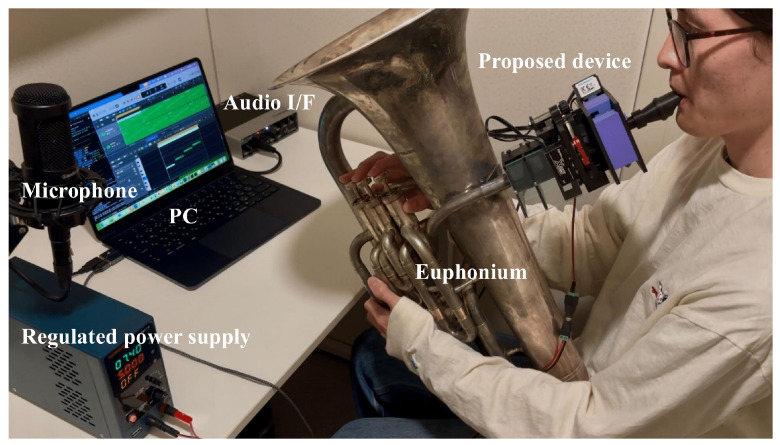
Experimental equipment and environment.

The equipment and experimental environment are summarized in Figure 12 and Table 3. All experiments were conducted in a soundproof room under controlled conditions: temperature 23–25 °C and humidity 35–45%.

### 3.2. Evaluation Criteria

The evaluation criteria for each experiment are as follows:

Exp. 1—Timbre Analysis: timbre was evaluated by recording the same target pitch (A♯3: 233.1 Hz) under two conditions—normal playing and playing with the device—and analyzing the waveform and spectrogram. From the normalized audio data, a stable-sounding interval (0.8–1.4 s) was extracted, and its spectrum was computed using the FFT. The recordings were stored as 48 kHz, 24-bit WAV files. Articulation performance was examined using the waveform and spectrogram, focusing on onset timing, harmonic strength, and amplitude variations produced by changes in blowing intensity.

Exp. 2—Pitch Range Test: The playable pitch range was evaluated by plotting the pitch transitions obtained for each fingering. When the natural frequency of the lips changes through position control, large pitch shifts (harmonic jumps) may occur without altering the fingering; the experiment verifies whether such transitions are feasible. For comparison with previous works, Section 4 discusses the present results alongside reference data from Gilbert et al.’s fully automatic trombone-playing system [9], since few prior studies report clear playable ranges for artificial buzzing devices. The trombone is a mid–low brass instrument with a range comparable to that of the euphonium.

Exp. 3—Pitch Stabilization Test: Experiment 3 evaluates the effectiveness of aperture control by comparing three conditions: Normal playing, Position control only, and combined Position + Aperture control. For each condition, the target pitch and the actual produced pitch (f0) were recorded as time-series data. Performance was assessed using both plotted results and the mean absolute error (MAE). Here, the purpose of pitch correction in this study is to correct the nonlinear behavior of the artificial lips. Since the desirable pitch stability is the ability to produce a pitch close to the target pitch, the mean absolute error between the estimated pitch and the target pitch was used as the evaluation method.

MAE was computed from data points where both the target pitch and f0 were available, using two metrics: frequency error and cent error within each pitch segment. The number of data points obtained in each pitch segment also serves as an indicator of system responsiveness. A larger number of valid points implies a shorter delay between sound onset and feedback initiation, leading to faster convergence toward the target pitch.

### 3.3. Results

The results of the experiment are as follows.

**Figure 13:**
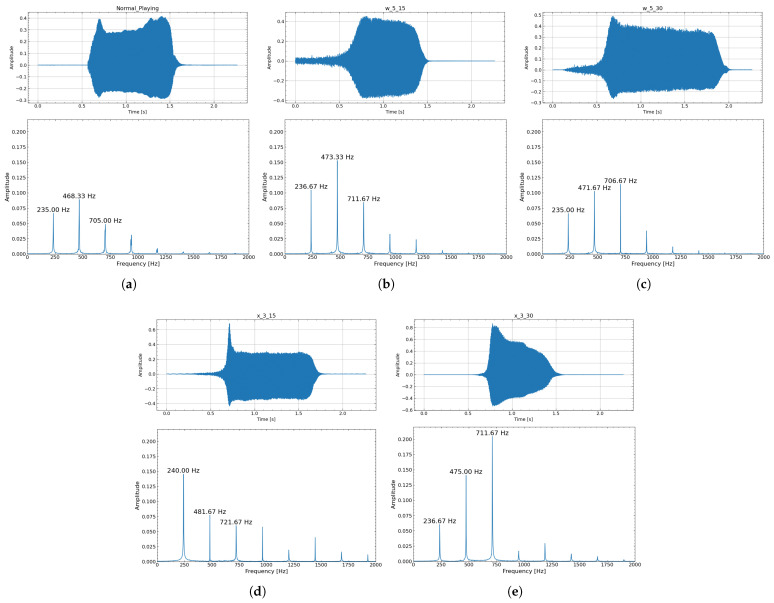
Exp. 1: Audio waveform and spectrum (0.8–1.4 s) when playing the target pitch A♯3 (233.1 Hz). (**a**) Normal playing. (**b**) Using w_5_15 Lip. (**c**) Using w_5_30 Lip. (**d**) Using x_3_15 Lip. (**e**) Using x_3_30 Lip.

**Figure 14:**
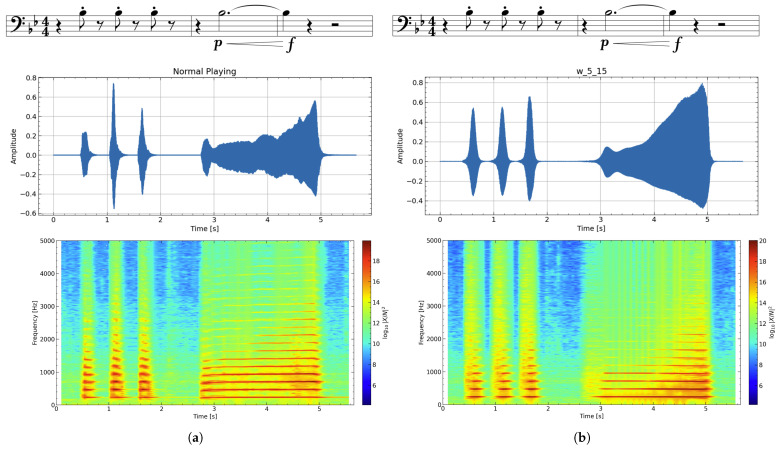
Exp. 1: Spectrograms of performance with articulation at the target pitch A♯3. The musical score shown above indicates the target articulation pattern, including staccato and crescendo. (**a**) Normal playing. (**b**) Using w_5_15 lip.

**Figure 15:**
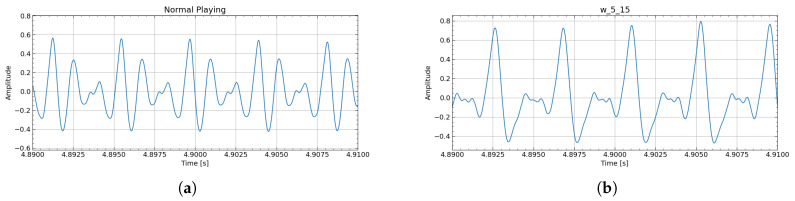
Exp. 1: Enlarged view of the performance audio waveform with articulation. Even with high blowing pressure and large amplitude, distortion-free acoustic characteristics can be achieved. (**a**) Normal playing. (**b**) Using w_5_15 lip.

Exp. 1—Timbre Analysis: The waveform and spectrum obtained when producing a single note A♯3 (233.1 Hz) in Experiment 1 are shown in Figure 13. For the articulation evaluation, the target score and the spectrograms of the actual performance sound (using w_5_15) are shown in Figure 14. Also, Figure 15 is an enlarged view of the high-amplitude part of the waveform.

**Figure 16:**
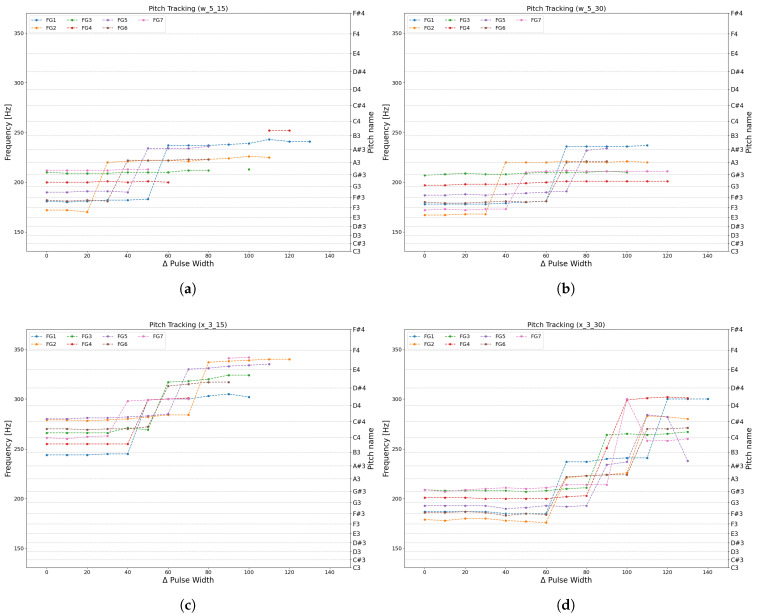
Exp. 2: Change in pitch that can be produced by position control. When the PWM value changes by 10, the artificial teeth moves forward by 0.5 mm. (**a**) Using w_5_15 Lip. (**b**) Using w_5_30 Lip. (**c**) Using x_3_15 Lip. (**d**) Using x_3_30 Lip.

Exp. 2—Pitch Range Test: The results of Exp. 2 are shown in Figure 16. The horizontal axis represents the change in PWM value used to control the rotation angle of the lip position control motor. The figure illustrates how the produced pitch varied across all fingerings as the motor position was incrementally adjusted. The final playable pitch ranges obtained for each fingering are summarized in Table 4.

**Figure 17:**
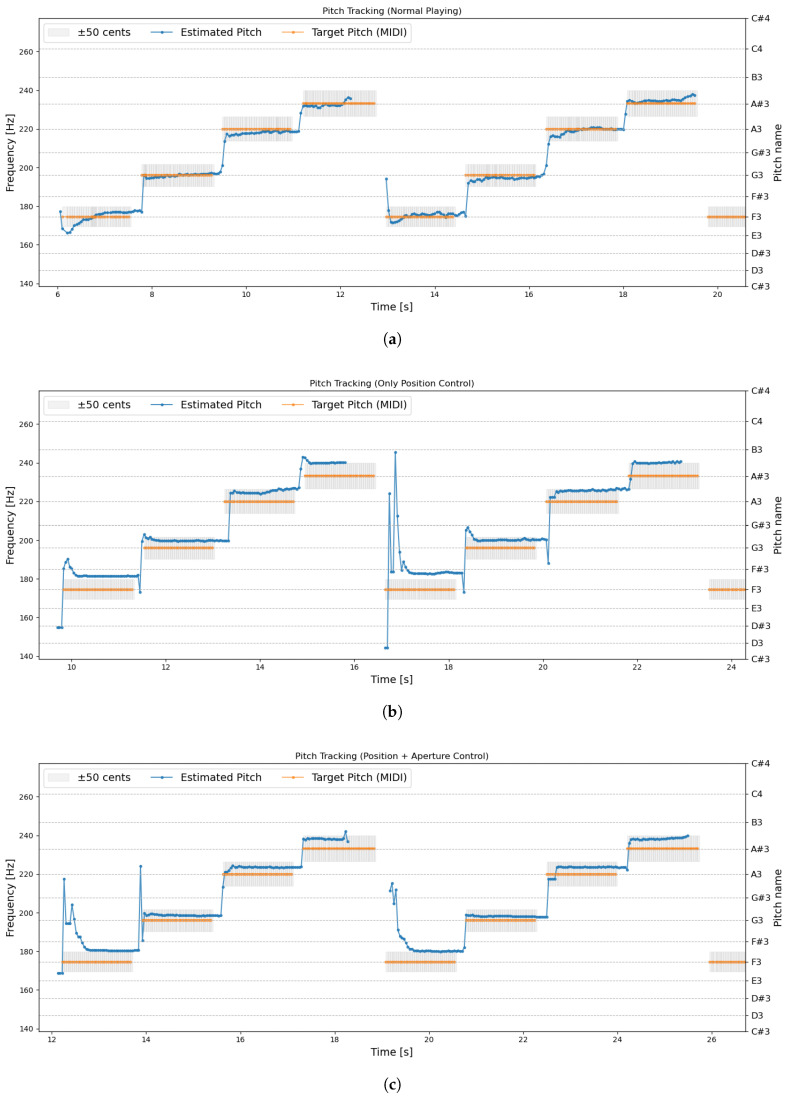
Exp. 3: Comparison of pitch stability under different control conditions. Aperture control reduces pitch deviation overall, particularly in steady-state regions. (**a**) Normal playing. (**b**) Position control only. (**c**) Position + Aperture control.

Exp. 3—Pitch Stabilization Test: Figure 17 shows the pitch transitions in Experiment 3, and Table 5 summarizes the number of valid pitch error samples and the resulting MAE values. The MAE of normal playing is presented as a reference. Figure 17a is normal playing without using the device, Figure 17b is position control only, and Figure 17c is position and aperture control. A video of the experiment is available via the link in Appendix A.

## 4. Discussion

This section discusses the effectiveness and characteristics of the proposed system based on the results obtained in Experiments 1–3.

### 4.1. Results of Artificial Lips Control System

#### 4.1.1. Exp. 1—Timbre Analysis

From the waveforms and spectra shown in Figure 13, self-sustained lip vibration was observed for all artificial lips, and acoustic characteristics similar to those of normal playing were obtained. Whereas normal playing allows rapid sound onset, device-assisted playing exhibited a tendency toward longer attack times. This phenomenon is described in further detail in Section 4.2.

In the spectral analysis, for some artificial lips, higher harmonics exceeded the fundamental, a typical feature of brass timbre in which the second harmonic helps sustain the fundamental standing wave [28]. Among all models, w_5_15 showed the closest harmonic structure to normal playing, with the second peak strongest, followed by the first and third.

Articulatory expressions such as staccato and crescendo were clearly observed in the device-assisted performance (Figure 14). Because the device uses the performer’s own airflow rather than a pump, expressive control can be applied in a manner close to normal playing. In the spectrogram, noninteger harmonics originate from airflow noise leaking through the lip aperture. Unlike normal playing, where intraoral pressure is released abruptly, the aperture remains open in the device, resulting in a nearly constant noise component.

Regarding timbre, w_5_15 showed the closest match to normal playing in both sustained and articulated sounds. As shown in Figure 15, even with increased blowing pressure—producing larger oscillation amplitudes—the waveform remains undistorted and the acoustic characteristics are maintained.

#### 4.1.2. Exp. 2—Pitch Range Test

From Figure 16, it is evident that multiple pitches can be produced with all artificial lips. There are many regions in which several pitches are generated at the same PWM value. This indicates that even without driving the position-control motor, a certain pitch range can be produced solely through valve (fingering) operation. Because fingering changes the resonant frequency of the instrument’s air column, the most easily excited resonance mode is preferentially sustained even when the natural lip vibration frequency remains unchanged. Regions in which the pitch rises abruptly with PWM adjustment can be observed, corresponding to harmonic jumps in which the oscillation mode transitions to a higher-order resonance. In this experiment, up to a third-harmonic jump was observed. Comparing the four artificial lips parameters, thicker lips tended to produce lower pitches more easily, while thinner lips facilitated the production of higher pitches. This result is consistent with previous findings showing that thicker lips are less likely to excite higher-order vibration modes [11].

For the x_3_30 artificial lips, increasing the motor displacement caused the pitch to momentarily jump to a higher mode before returning. This likely reflects increased sensitivity of the oscillation conditions due to changes in lip geometry and tension. Because the system operated near the minimum blowing pressure for phonation, small variations allowed it to settle at the most resonant frequency, resulting in these mode shifts. These results suggest that position control alone cannot fully stabilize the regime, and that adding aperture control would improve robustness.

The pitch ranges obtained with the proposed system are summarized in Table 6. Although the full euphonium range was not reached, a single-actuator artificial lips mechanism achieved a pitch variation of nine semitones, comparable to fully automated trombone-playing devices, demonstrating practical capability despite its simple structure. The system also allows quick replacement of artificial lips, enabling users to choose thicker models for lower pitches and thinner ones for higher pitches—an important advantage in adapting the device to different performance needs.

#### 4.1.3. Exp. 3—Pitch Stabilization Test

The results of Experiment 3 demonstrated that the system can operate properly even during repeated position-control movements. In particular, because F3 and A♯3 share the same fingering, the observed pitch transitions indicate harmonic jumps triggered purely by position control, corresponding to changes in the excited resonance mode. The graph shows that compared to normal playing (a), using the device (b, c) shows less fluctuation in pitch within a single note. This is thought to be because, unlike normal playing where the performer maintains the lip position at each moment, the artificial lips pressure position is fixed using position control. Performance sounds, such as fluctuations and pitch bending, usually vary depending on the performer, whereas the use of the device suggests the possibility of producing steadier and more stable tones.

Regarding control responsiveness, Table 5 shows that about 260 samples were obtained. The 11.6 s duration of the target-pitch segments corresponds to an update interval of roughly 44 ms. Thus, the system can follow pitch transitions with sufficiently low latency.

With respect to pitch stability, the introduction of aperture control resulted in a clear reduction in pitch error compared with position control alone. Because the interval between adjacent semitones is 100 cent, values within ±50 cent can be considered to correspond to the intended pitch. From the MAE results, the cent-based pitch error decreased by approximately 22% when aperture control was applied, supporting its effectiveness in stabilizing the produced pitch.

As shown by the pitch trajectories in Figure 17b,c, the produced pitch approaches the target pitch more closely overall. Although this effect is not always evident during some onset phases, it is clearly reflected in the reduced pitch error during the steady-state segments of each note. Furthermore, when returning to F3 (the fifth note in the sequence), the position-only control condition exhibits large pitch fluctuations. This behavior is likely caused by hysteresis inherent in elastic materials such as artificial lips, where tension and geometry do not immediately return to their initial state. In contrast, when aperture control is combined with position control, this delay is compensated, resulting in faster convergence to the target pitch.

Across all conditions, the produced pitch tended to be slightly higher than the target. This likely results from changes in the instrument’s resonant frequency caused by attaching the device, a shift common to wearable mechanisms. If more precise pitch is required, the instrument should be re-tuned with the device attached. Compared to normal playing, differences in pitch behavior remain, and further development will be required to better reproduce the characteristics of human performance.

### 4.2. Limitation

The experimental results demonstrated that the proposed device can reproduce key aspects of brass instrument sound production with controllable timbre and pitch. However, limitations related to the device’s physical and structural design were also identified and are discussed below.

#### 4.2.1. Exp. 1: Attack Time

**Figure 18:**
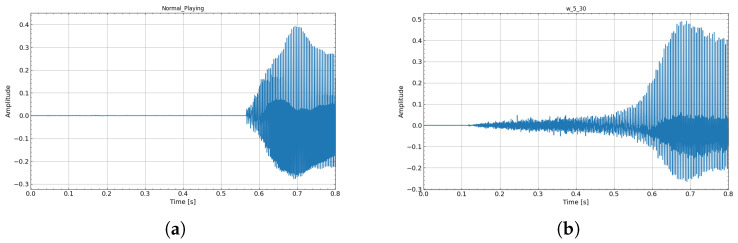
Limitation 1: Comparison of attack time in Experiment 1. Thicker lips with larger air holes show a longer rise time. (**a**) Normal playing. (**b**) Using w_5_30 lip.

Figure 18 enlarges the attack portion of the recorded waveforms. Because the proposed artificial lips include an air aperture, part of the airflow escapes before reaching the instrument, making it difficult for intraoral pressure to rise rapidly. Consequently, the attack time tends to be longer than in normal playing. A possible solution is to temporarily close the air aperture before phonation to allow pressure to build up, and then open it immediately when the required threshold airflow is detected.

#### 4.2.2. Exp. 2: Relationship Between Experimental Results and Lip Vibration Model

Although the device was designed based on a model in which the oscillation frequency increases as the lips are pushed toward the mouthpiece, Exp. 2 showed that a full one-octave pitch shift could not be achieved. This may be because the lips had to be clamped firmly to maintain airtightness, resulting in non-negligible tension even at the initial position, which likely deviated from the simple-support condition assumed in the model. Consequently, higher pitches were easier to produce, whereas lower pitches were more difficult.

### 4.3. Future Work

Building on the current system, future work will extend the functionality of the artificial-lip device through the following directions.

(a) Development of a control model and parameter optimization
  The current system assumes a monotonic relation between lip position, aperture, and oscillation frequency, but in practice factors such as lip material introduce more complex behavior, leaving room for further optimization. Constructing a more realistic control model—e.g., incorporating breath pressure retention—and identifying optimal parameters would improve reproducibility and operability. Future work may also include measuring steady-state blowing pressure by integrating a pressure sensor, which may contribute to a more detailed characterization of the device behavior.
(b) Mitigation of humidity effects from human breath
  The device does not account for humidity changes inside the chamber, and the viscoelastic artificial lips are sensitive to moisture, which may affect vibration during long-term use. Adding humidity shielding or sensing and compensation mechanisms would help maintain stable performance.
(c) Considerations for acoustic research applications
  In the present experiments, sound was recorded using a condenser microphone placed approximately 30 cm from the bell of the instrument (Figure 12). The microphone position and playing posture were kept as consistent as possible during recording, although the setup was not mechanically fixed.
  We consider this experimental environment to be acceptable for the comparative evaluations presented in this study; however, more strictly controlled conditions will be required to further develop the proposed device for acoustic research. Future work will focus on establishing more rigorous and standardized experimental setups, including improved fixation of the instrument and microphone. Such developments will enable more reproducible measurements and facilitate comparative analysis of instrument-specific acoustic properties.
(d) Musical performance and novel music experiences
  If the stability of artificial lips control is further improved, musical performance becomes feasible. Faster attack response and a wider pitch range will be essential for melodic playing. In addition, further development of the pitch correction system could enable more flexible pitch control, such as controlled pitch bending or adaptation to different tuning systems, thereby enriching musical expression. Achieving these capabilities could enable the reproduction of human-like performance characteristics and facilitate novel musical experiences.

## 5. Conclusions

In this study, we proposed an attachable semi-automatic artificial lips control device for brass instruments and experimentally demonstrated its effectiveness. By combining lip-pushing position control with wire-based aperture control, the device enables a human performer to generate brass sound without relying on their own lip vibration. Stable pitch control is achieved through a combination of pre-calibration and real-time pitch feedback, with sufficient actuation accuracy in a compact and lightweight design.

Timbre evaluation confirmed that the device can produce realistic brass tones with harmonic structures comparable to normal playing and can reproduce basic articulations. Experiments with multiple artificial-lips types showed that the system covers a practical pitch range and supports pitch transitions, including harmonic jumps, with up to nine semitones of pitch variation using a single lip model. In addition, real-time acoustic sensing enabled compensation of pitch deviations, resulting in more stable sound production, with a 22% reduction in MAE.

Rather than replacing human performers with a fully automated mechanism, the proposed system provides a simple, controllable, and reproducible framework for brass sound generation and pitch stabilization, serving as a platform for semi-automatic brass-playing robotic systems and related experimental studies.

Based on the experimental results, the assumed use cases of the device and its current limitations are summarized in Table 7, clarifying its applicability to robotic performance, acoustic experiments, and musical experience-oriented use, as well as the constraints to be addressed in future work.

## Figures and Tables

**Table 1:** Fingering number table (pistons to be pressed are indicated by ∘).

Fingering Number	1st Valve	2nd Valve	3rd Valve
FG1			
FG2		∘	
FG3	∘		
FG4			∘
FG5		∘	∘
FG6	∘		∘
FG7	∘	∘	∘

**Table 2:** Artificial lips parameters. “w” indicates that the artificial lips are wired, while the two 3 mm thick artificial lips are not wired because of their thinness.

Parameter	Wire	Thickness [mm]	Air hole [mm]
w_5_15	∘	5	1 × 15
w_5_30	∘	5	1 × 30
x_3_15	×	3	1 × 15
x_3_30	×	3	1 × 30

**Table 3:** Equipment used in the experiment. (^†^ Used only in Experiment 2).

Parts	Manufacturer	Product Name
Euphonium	PLAYTECH (Tokyo, Japan)	PTEP-300SS
Mouthpiece	YAMAHA (Hamamatsu, Japan)	SL-48DL
Microphone	Audio-Technica (Tokyo, Japan)	AT2035
Audio Interface	Steinberg (Hamburg, Germany)	UR22mkII
PC	Apple (Cupertino, CA, USA)	MacBook Air/macOS Sequoia 15.1
DAW	Apple (Cupertino, CA, USA)	Logic Pro 11.2.2
Pitch Identification Tool ^†^	TonalEnergy (Dallas, TX, USA)	TonalEnergy Tuner & Metronome app

**Table 4:** Exp. 2: Pitch ranges for each pair of artificial lips.

Artificial Lips	Frequency [Hz]	Pitch Name	Number of Semitones
w_5_15	172–252	F3–B3	6
w_5_30	167–237	E3–A♯3	6
x_3_15	244–340	B3–F4	6
x_3_30	179–300	F3–D4	9

**Table 5:** Exp. 3: Comparison of pitch error between normal playing, only position control, and position and aperture control. The cent-based MAE decreased by about 22% when aperture control was applied.

Method	Number of Data	MAE [Hz]	MAE [Cent]
Normal Playing	267	1.9	16.4
Position Control	259	7.2	62.1
Position + Aperture Control	262	5.6	48.3

**Table 6:** Discussion: Comparison with pitch ranges obtained using other methods.(Experiment 2).

Method	Frequency [Hz]	Pitch Name	Number of Semitones
Normal range (Euphonium)	77.8–466.2	D♯2–A♯4	31
Gilbert [9] (Trombone)	169–290	E3–D4	10
Proposed system (One pair of artificial lips)	179–300	F3–D4	9
Proposed system (Total)	167–340	E3–F4	13

**Table 7:** Assumed use cases of the proposed device, along with its helpful properties and potential limitations.

Assumed Use Case	Helpful Properties of the Proposed Device	Limitations
Semi-automatic brass-playing robots	Simple and compact sound generation mechanism using a real brass instrument; sound production without requiring buzzing by the performer’s lips; stable pitch generation via artificial lips control.	Limited expressive control compared to human performance; restricted pitch range and attack response.
Preliminary and comparative acoustic research	Ability to generate sounds from real brass instruments without requiring an experienced performer; easy setup compared to large conventional automatic blowing systems.	Experimental conditions are not yet fully standardized; stricter experimental control (e.g., mechanical fixation) is required for higher reproducibility.
Musical performance and novel music experiences	Ability to generate sounds without prior playing experience; stable sustained tones that allow users to easily obtain a musical experience; support for pitch transitions including harmonic jumps and basic articulations.	Expressive control is limited to stable pitch generation; musical expression using the device cannot be regarded as natural human-like performance.

## Data Availability

The original contributions presented in this study are included in the article. Further inquiries can be directed to the corresponding author.

## Appendix A. Video of Performance Using the System (Experiment 3)

The video below shows the author playing the euphonium using the proposed system. https://youtu.be/LRc8_8zh8HM, (accessed on 1 February 2026).
